# Molecular Insights Into the Relationship Between Autoimmune Thyroid Diseases and Breast Cancer: A Critical Perspective on Autoimmunity and ER Stress

**DOI:** 10.3389/fimmu.2019.00344

**Published:** 2019-03-01

**Authors:** Safikur Rahman, Ayyagari Archana, Arif Tasleem Jan, Durgashree Dutta, Abhishek Shankar, Jihoe Kim, Rinki Minakshi

**Affiliations:** ^1^Department of Medical Biotechnology, Yeungnam University, Gyeongsan, South Korea; ^2^Department of Microbiology, Swami Shraddhanand College, University of Delhi, New Delhi, India; ^3^School of Biosciences and Biotechnology, Baba Ghulam Shah Badshah University, Rajouri, India; ^4^Department of Biochemistry, Jan Nayak Chaudhary Devilal Dental College, Sirsa, India; ^5^Department of Preventive Oncology, Dr. B. R. Ambedkar Institute Rotary Cancer Hospital, All India Institute of Medical Sciences, New Delhi, India

**Keywords:** lymphocytic infiltration, Grave's disease, Hashimotos's thyroiditis, autoantigens, autoantibodies

## Abstract

The etiopathologies behind autoimmune thyroid diseases (AITDs) unravel misbehavior of immune components leading to the corruption of immune homeostasis where thyroid autoantigens turn foe to the self. In AITDs lymphocytic infiltration in the thyroid shows up a deranged immune system charging the follicular cells of the thyroid gland (thyrocytes) leading to the condition of either hyperthyroidism or hypothyroidism. The inflammation in AITDs consistently associate with ER function due to which disturbances in the ER protein homeostasis leads to unfolded protein response (UPR) that promotes pathogenesis of autoimmunity. The roles of ER stress in the instantaneous downregulation of MHC class I molecules on thyrocytes and the relevance of IFN γ in the pathogenesis of AITD has been well-documented. Thyroglobulin being the major target of autoantibodies in most of the AITDs is because of its unusual processing in the ER. Autoimmune disorders display a conglomeration of ER stress-induced UPR activated molecules. Several epidemiological data highlight the preponderance of AITDs in women as well as its concurrence with breast cancer. Both being an active glandular system displaying endocrine activity, thyroid as well as breast tissue show various commonalities in the expression pattern of heterogenous molecules that not only participate in the normal functioning but at the same time share the blame during disease establishment. Studies on the development and progression of breast carcinoma display a deranged and uncontrolled immune response, which is meticulously exploited during tumor metastasis. The molecular crosstalks between AITDs and breast tumor microenvironment rely on active participation of immune cells. The induction of ER stress by Tunicamycin advocates to provide a model for cancer therapy by intervening glycosylation. Therefore, this review attempts to showcase the molecules that are involved in feeding up the relationship between breast carcinoma and AITDs.

## Introduction

The eminent Nobel Laureate Paul Ehrlich predicted the concept of autoimmunity, calling it “horror autotoxicus” involving a perplexing situation wherein the immune system starts assaulting self-cells leading to the development of autoimmune diseases. Today autoimmune diseases pose notable clinical issues owing to their chronic nature and higher rate of prevalence in relatively younger populations who are at the peak of their reproductive years (1). Immunological self-tolerance, a seminal selection strategy wherein immune system prevents the reaction of lymphocytes with self-antigens, involves a wide array of genes and their cellular expression patterns, which upon disruption by inherited mutations or environmental factors result in autoimmune diseases. The characteristic feature of autoimmunity is the development and propagation of auto reactive T lymphocytes and autoantibodies against body's autoantigens. Such autoimmune diseases can be systemic like systemic lupus erythromatosus (SLE) or organ-specific as in the case of autoimmune thyroid diseases (AITDs) (2). In AITDs, lymphocytic infiltration in the thyroid shows up a deranged immune system charging the follicular cells of the thyroid gland (thyrocytes) leading to the condition of either hyperthyroidism or hypothyroidism. The organ-specific autoimmune attack on thyroid witnesses inflammation of the thyroid tissue leading to thyrotoxicosis [Reviewed in (3)]. Extensive study reports on AITDs show two major clinical manifestations of thyroid autoimmunity, Hashimoto's thyroiditis (HT) (most common cause of hypothyroidism) and Grave's disease (GD) (most common cause of hyperthyroidism). The epidemiological data on the preponderance of AITDs in women is intriguing [Reviewed in (4)]. It is noteworthy that the occurrence of hyperthyroidism, caused by GD as well as hypothyroidism in HT, is 10 times more in women as compared to men [Reviewed in (4)]. So, it becomes important here to rekindle the higher prevalence rate of AITDs in women. Congruent to this idea, there is an emerging repertoire of evidences that show relationship between AITDs and breast cancer in women (5).

Hence, the fulcrum of the present review is an attempt to delineate the molecules, which are involved in crosstalks between two major AITDs (GD and HT) and breast cancer pathology.

## The Autoantigens of Thyroid Gland

**Figure 1:**
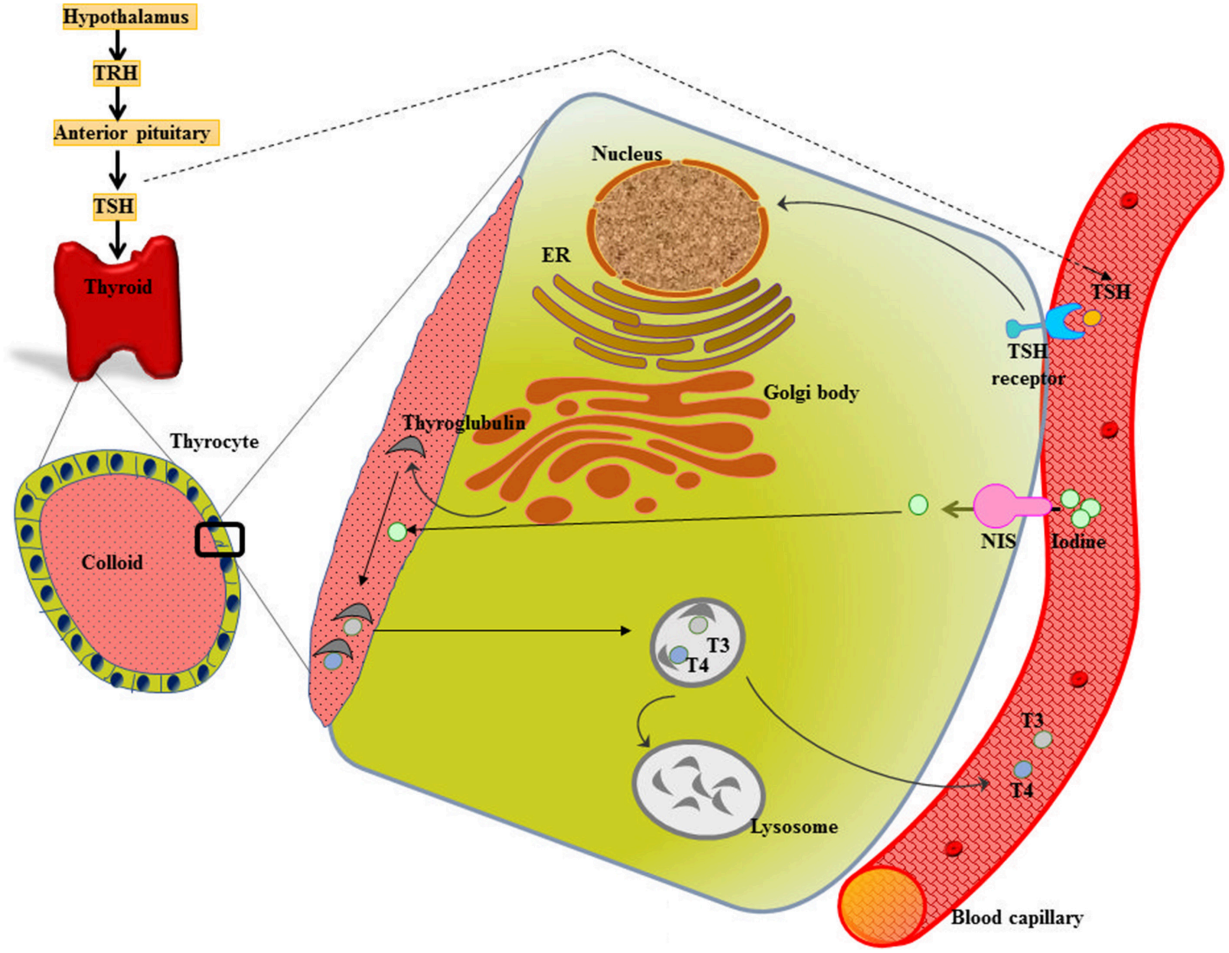
Thyroid gland function and structure. The hypothalamus secretes thyrotropin releasing hormone (TRH) that stimulates the anterior pituitary to release thyroid stimulating hormone (TSH). TSH acts on the TSH receptor (TSH-R) leading majorly to the activation of two crucial steps-expression of sodium/iodide symporter (NIS) that functions in uptake of iodine into the thyrocyte from the blood stream and biosynthesis as well as release of thyroid hormones T3 and T4.

Metabolic homeostasis is much under the control of a properly functioning thyroid gland. The two major thyroid hormones are L-thyroxine (tetraiodothyronine, T4) and L-triiodothyronine (T3), release under the influence of thyroid stimulating hormone (TSH). TSH acts on TSH receptors (TSH-R) (expressed all over the basolateral membrane of thyrocytes) and stimulates the expression of membrane protein called sodium/iodide symporter (NIS), which mediate the inward translocation of iodine into the thyrocytes. [Reviewed in (6, 7)] (Figure 1).

The spectrum of autoantigens reacting with the patient sera in cases of AITDs mainly involves three thyroid autoantigens: thyroglobulin (Tg), thyroid peroxidase (TPO), and TSH-R. These complex glycosylated proteins are post-translationally modified that affect their stimulatory role on the immune system.

### Thyroglobulin (Tg)

The most abundant glycosylated iodoprotein in the thyrocyte lumen is Tg (200–300 g/l), which is 670 kDa in size. Tg, accumulated in the colloid, provides the matrix for the sequestration of iodine and synthesis of thyroid hormones [Reviewed in (8)]. Tg iodination makes it a prohormone of T4 and T3. Tg antibodies majorly recognize the immunodominant epitopes on Tg molecule known as region II in GD or HT patients (9). Anti-Tg polyclonal antibodies have been reported in 30% of GD patients. The cell surface expression of Tg is regulated by TSH. The Tg gene sequencing studies showed that variations in the amino acid sequence is associated with AITDs (10).

### Thyroid Peroxidase (TPO)

TPO is germane to enzymatic action of iodination of Tg in the presence of H_2_O_2_ and synthesis of thyroid hormones. Owing to its intracellular localization, it is also known as “microsomal antigen” [Reviewed in (11)]. Over 90% of patients with GD show the presence of anti-TPO autoantibodies (12).

### TSH Receptor (TSH-R)

TSH-R is a member of G-protein coupled receptor family with seven transmembrane domains. Accumulating evidences support the presence of anti TSH-R autoantibodies in GD patients [Reviewed in (8)]. The receptor is a glycoprotein of 764 amino acids, which shows the architectural pattern of an extra-membranous region (A subunit) tethered through seven transmembrane loops into the membrane that ends into an intracellular domain (B subunit) showing association with G_s_ subunit of adenyl cyclase (13). TSH-R shows a remarkable feature of undergoing post-translational cleavage wherein the holoreceptor loses the C-peptide region of the polypeptide chain (A subunit), which has been proven to potentiate autoimmune response (14, 15).

Apart from the above-mentioned autoantigens, NIS has been accepted to be the fourth important autoantigen in AITDs. It has been documented that 1/3rd of sera from GD and 15% sera from HT patients contain NIS inhibiting antibodies (16). Studies have shown that iodide uptake is inhibited by anti-NIS antibodies that affect the function of the thyroid gland (17).

### The Synthesis of Thyroid Hormones

The iodide from blood capillary is taken up into the thyrocyte by NIS, which is followed by its transport across the cell and finally efflux into the follicular lumen (colloid). The expression of Tg genes leads to the secretion of Tg into the colloid, where Tg serves as a scaffold for the synthesis of thyroid hormones. After iodide comes on the thyrocyte-colloid interface, it gets oxidized and is incorporated into Tg enzymatically by TPO. Following a sequence of reactions, described elsewhere, TPO catalyzes the synthesis of T3 or T4 (18). The release of thyroid hormones into the bloodstream is through pinocytosis (18) (Figure 1).

## The Rogue Lymphocytes in Autoimmune Thyroid Diseases (AITDs)

The self-non-self discrimination ability of the immune system has well-evolved not only to protect us against invading foreign proteins (non-self-antigens) but also to tolerate the self-antigens. However, it has been reported that in 20% of adult women, antibodies to thyroid antigens are present and this situation is clinically considered normal. In the pathophysiology of AITDs, both humoral (antibody) as well as cell-mediated mechanisms assault thyroid cells.

### Immunogenicity of Thyroid Autoantigens

**Figure 2:**
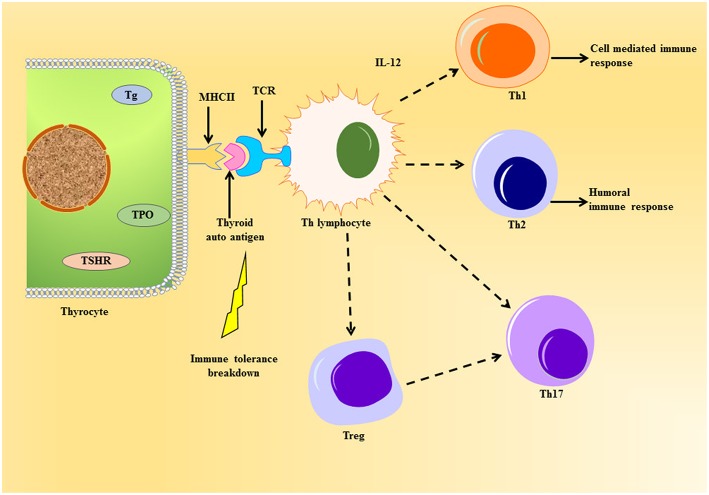
Schematic representation of thyroid autoantigen presentation on the thyrocyte leading to the activation of T cell cascade. The thyrocytes can act as non-professional antigen presenting cell, whereby they can present thyroid autoantigens to Th lymphocyte during autoimmune response. This event can elicit the activation of various T cells like the Tregs, Th 17, and the Th subsets, Th1 and Th2, under the influence of cytokines released. Unlike the normal immune response, the immune cells violate homeostasis during the emergence of thyroid autoimmunity imposed by the genetic and environmental factors.

The professional antigen presenting cells (APC) endocytose proteins, process and present them as peptides on their surface major histocompatibility complex class II (MHC class II) protein to T cell antigen receptor (TCR) of naïve CD4^+^T helper (Th) lymphocytes. The thyrocytes or the thyroid follicular cells (TFC), being polarized epithelial cells, show endocytic activity by internalizing colloid rich in thyroglobulin to exocytose generated thyroid hormones. TFC become non-professional APCs leading to the presentation of thyroid autoantigens on MHC class II (19). Tg protein, being large and abundant in the thyroid, is largely presented on MHC class II protein (20) (Figure 2). Apart from the size and availability of autoantigens, their glycosylation status plays an important role in T-cell response (21).

The cells can further cause surge in thyroid infiltration or help in the differentiation of cytotoxic T cells (CTL). There are reports of a unique subset of Th cell called Th17 lymphocyte, which characteristically produce IL-17 causing exacerbation of autoimmune response (22, 23). The main role of Th17 has been seen in causing inflammatory tissue damage during autoimmune response (24). The differentiation of Th17 is dependent on intracellular pathways like signal transducer and activators of transcription-3 (STAT3) and other cytokines (24).

Another important subset of CD4^+^T lymphocyte worth mentioning is T regulatory cells (Tregs), which have CD25 protein and IL-2 receptor on their cell surface (25). They express forkhead box P3 (FoxP3) gene, a transcription factor and specific marker for Tregs, which has been known to undergo mutation leading to the development of AITD (26). They have been well-addressed in AITDs (27) and are known to maintain tolerance through suppression of self-reactive T cell activation (28). Worth mentioning here is the transmembrane protein CTLA-4 (or CD152), which is constitutively expressed in FoxP3^+^ Tregs, have been known for suppressing T cell response thereby acting crucial in the sustenance of tolerance to self-antigens [Reviewed in (29)]. Thus, Th17 and Tregs oppose each other in the pathophysiology of AITDs, thereby proving the importance of measurement of Th17/Treg in the development of such maladies (30).

### Role of Natural Killer (NK) Cells and Macrophages

NK cells release cytokines and kill abnormal/foreign cells by varied mechanisms (31). The cytokines released by NK cells serve to activate macrophages. In the process called antibody-dependent-cell-cytotoxicity (ADCC), the macrophages and NK cells kill non-self-cells/altered self-cells coated with immune complexes (32). The state of exacerbated thyrotoxicity in both GD and HT may be attributed to a hike in the NK cell activity (33).

## Molecules Participating in the Destruction of Thyroid

The role of apoptosis in the maintenance of cellular homeostasis is very important in the proper functioning of the body. Coclet et al. approximately calculated that during a lifetime the turnover of thyroid gland is ~5 years (34). Reports suggest that normally the apoptosis of thyrocytes is maintained at low levels but goes up in various cases of thyroiditis (35). The lymphocytic infiltration of thyroid in AITDs initiate cell-mediated cell death. Under the conditions of prevailing inflammation, the surface expression of Fas ligand (FasL or CD95L) and production of Fas (hence the upregulation of Fas signaling cascade) on/by both thyrocytes as well as activated T cells have been finely documented in AITD (36, 37). The cytotoxic effector cells, CTL and NK cells show constitutive expression of FasL [Reviewed in (38)]. Reports suggest that there is a constitutive expression of Fas mRNA in normal thyrocyte but, expression of Fas protein is evidenced only after lymphocytic infiltration in the inflammation foci of the thyroid gland (37). The apoptosis mediated by Fas in the thyrocytes requires their induction through IFN-γ and TNF-α [Reviewed in (36)].

The TNF-related apoptosis-inducing ligand (TRAIL) shares homology with FasL. The receptors for TRAIL, Death Receptor 4 (DR4), and Death Receptor 5 (DR5) have been shown to be expressed in the thyrocytes under the stimulatory effects of IFN-γ in blend with TNF-α or IL-1β (39). The infiltrating lymphocytes also showed significant concentrations of TRAIL mRNA in the thyrocytes (39).

### Molecules Aiding in Lymphocytic Infiltration of the Thyroid Gland

The role of adhesion molecules in the migration of lymphocytic cells to the thyroid gland is indispensable. A series of adhesive molecules participate in the extravasation of lymphocytes through the endothelial cells. The expression of selectins and integrins on the endothelium forms a critical stage in the accumulation of lymphocytes in thyroid gland. There is a surge in the expression of key molecules involved in lymphocytic attachment to endothelium in AITDs, these molecules are: the β1-integrins (mediating the cell attachment to the extracellular matrix proteins), vascular cell adhesion molecule-1 (VCAM-1) and intercellular adhesion molecule-1 (ICAM-1) (40). For example, T cells show higher production of IFN-γ, IL-1 and TNF-α, which potentiates the expression of adhesion molecule receptors thereby augmenting autoimmune response (41). Interestingly, AITD associated rise in the vasculature of the intra-thyroid environment is in coherence with the high concentrations of vascular endothelial growth factor (VEGF), angiopoietins (Ang-1 and Ang-2) and tyrosine kinase receptor Tie-2 family signaling molecules (important for angiogenesis and vascularization) (42). There are reports of monocytes expressing Tie-2 that are recruited to inflammation foci or neoplastic center (43). All these events compound up in causing tissue damage evident in AITD (42).

## Causes of AITDs

The genetic susceptibility to AITD encompasses genes coding for molecules participating in thyroid function and immune response. The reviews describing genes that are susceptible to AITD are discussed elsewhere (44). We have reports from epidemiological studies that give convincing evidence about the genetic predisposition of AITDs in samples of familial clustering, female candidates and twins (45). One study on whole-genome screening of multiplex families, identified unique susceptibility loci for AITDs (46). Genetic factors predisposing candidates to HT have also been studied (47). Approximately 80% of AITDs occur due to genetic factors, and the remaining contribution is due to environmental triggers. Exposure to radiation has been proven to trigger AITDs. Epidemiological studies define the dietary intake of iodine as one of the environmental triggers for HT. Stress has also been reported to cause GD onset. Infections due to bacteria as well as virus and the condition of pregnancy are other triggers of HT [Reviewed in (48)].

Similarly, serological studies presented evidences in favor of bacterial as well as viral infections in GD predisposition (49). The active contribution of epigenetics in the development of AITD has been well-addressed (50).

## The Case of GD

The pathophysiology of GD comprises thyroid growth, incessant thyroid hormone production and orbital inflammation leading to ophthalmopathy (51).

### Interplay of Immune Cells and Other Molecules in GD

TSH-R is the main autoantigen in GD that breaks immune tolerance. Inside the thyrocyte, TSH-R precursor is subjected to post-translational intramolecular proteolysis at multiple sites to remove an intervening polypeptide stretch, giving rise to a 2-subunit receptor structure, the α or A subunit (TSHR A) and the β or B subunit (TSHR B). The two subunits are linked together with disulphide bonds, which upon reduction release the α subunit (ectodomain) (this phenomenon is called “receptor shedding”) from its membrane-anchored receptor subunit β (52). Studies on AITDs have shown that thyrocytes display the feature of processing thyroid autoantigens on MHC class II complex, which is certainly considered an inappropriate act of thyrocytes (because they are not inherently an APC) (53). So, a study hypothesized that this inappropriate expression of MHC class II complex on thyrocytes enhances their ability to present thyroid autoantigens to Th cells thereby potentiating autoimmune response (54). Collective data from various assays on the insight into the involvement of Th subpopulation in GD clearly proves the role of Th1 in the malady (29). With respect to Tregs, variants of CTLA-4 genes have been reported in GD patients (55). In a remarkable study on cells obtained via fine-needle aspiration biopsy from GD patients, it was shown that the T cells in thyroid infiltration tested positive for *in vitro* cell-mediated immunity (CMI) (56). The *in vitro* analysis of the expression of MHC class II complex and presentation of thyroid autoantigen on thyrocytes to T cells, conclusively shows the involvement of IFN-γ in this event (in GD patient sample) (57).

The anti-TSHR antibodies (TRAb) [belonging to immunoglobulin (Ig) G class] directed against TSH-R has been categorically described into three types: (i) thyroid stimulating antibody (TSAb) or thyroid stimulating immunoglobulin (TSI): binds to an epitope on TSH-R leading to the activation of the receptor (the effect is same as that of TSH), (ii) thyrotropin binding inhibitory immunoglobulins (TBII) or thyroid stimulation blocking antibody (TSBAb): bind to same or different epitope where they obstruct binding of radiolabelled TSH (shown in assays), and (iii) neutral TRAb. The physio-pathological development of GD observes unregulated and incessantly functional thyroid cells that are stimulated by TSAb (58). It has been proven *in vitro* analysis that the free α subunit of TSH-R (the self-antigen) is preferentially neutralized by autoantibody due to its free accessibility thereby cause ascension in autoimmune response manifesting GD (59). The binding of TSAb to the ectodomain of TSH-R activates cAMP signaling that stimulates the unregulated production of thyroid hormones leading to hyperthyroidism. This shows biochemical features of GD with high levels of thyroid hormones and low to almost undetectable concentrations of TSH (60). The high titer of TSAb against TSH-R is the characteristic feature of GD, wherein TSAb activates TSH-R in the absence of TSH (61). In 95% of GD patient samples, TSAb was a notable candidate (62). In a remarkable study by Pichurin et al. it was shown that the presentation of endogenously expressed and processed TSH-R on MHC class II protein of thyrocyte significantly induced T cell response and TSAb, symptomizing the Graves' hyperthyroidism in mice (63). Furthermore, experiments in animal models, endogenous processing and presentation of TSHR A showed higher induction of TSAb as compared to non-cleaving TSH-R resulting in GD (64). In most of the studies reported on GD patients, TPO autoantibodies are frequently detected (65).

The persistence of GD is strongly exerted by the presence of B cells in thyroid lymphocytic infiltration. This was proven by showing beneficial effect of depleting B-cells by the antibody rituximab in GD (66). Apart from the competence of antibody production, B cell-mediated immunosuppressive roles have also been elucidated. The sporadic occurrence of the population of immunoregulatory B cells (Bregs) in GD has been suggested (67). Bregs have been identified to secrete IL-10, which is anti-inflammatory thereby aiding in the sustenance of immune tolerance. Alterations in function and concentration of Bregs have been observed in autoimmune diseases where severity of the disease shares inverse correlations with Breg status (68). IL-10 has an inhibitory role in APCs, where it impedes the secretion of TNF-α; mitigates surface expression of MHC class II complex and adhesion molecules. Also, Bregs directly affect the CD4^+^T cell differentiation (69).

GD patient serum shows elevated levels of ICAM-1, VCAM-1, and E-selectins that are all important in aiding the lymphocytic infiltration of the thyroid gland, which is a reflection of the persistent autoimmune response (70). Additionally, Grave's patients with ophthalmology manifest have been studied to show higher serum levels of soluble ICAM-1 (sICAM-1) (71).

There are high serum levels of soluble Tie-2 and Ang-2 in GD patients, which supplement the progression of hyperplasia (angiogenesis) and inflammation (42).

### Effect of Autoimmunity Beyond Thyroid in GD

Albeit GD patients show tremendous immunomodulatory assault of the thyroid gland, the extrathyroidal embodiment of the disease holds its enigmatic effect. There are reports of orbital infiltration leading to inflammation and connective tissue remodeling in the orbit that is the manifestation of thyroid-associated ophthalmopathy (TAO) (51). The involvement of autoimmunity to TSH-R has been proposed in the TAO foci (72). In a study by Pritchard et al. it was observed that IgG mediated activation and secretion of the two strong T cell chemo attractants, RANTES (CCL5) and IL-16, from fibroblasts proved the role of these chemoattractants in the T cell infiltration of the thyrocytes (73). Besides, studies also report about the reinforced production of collagen in human fibroblasts under the effect of IgG against TSH-R (74). Reports implicate about the participation of fibrocytes, the progenitor cells of monocyte, in thyroid as well as orbit infiltration (75). Adding to the continuum of molecules participating in the story of thyroid autoimmunity of GD, the partaken by insulin-like growth factor-1 receptor (IGF-1R), which is stimulated by IgG, need a special mention here (76).

Showing broad expression pattern in various tissues, IGF-1R, a tyrosine kinase with extracellular and membrane-spanning subunits, gets activated by growth factor ligands, IGF-1 and IGF-2 to start cellular proliferation and antagonize apoptosis (77). Overexpressing IGF-R1 elicits malignant transformation (78). Apart from the aforementioned ligands of IGF-1R, there are other proteins that bind to the receptor under hormonal control, which are collectively called insulin-like growth factor-1 binding protein (IGFBP). One such example is the regulation of IGFBP (specifically IGFBP-3) synthesis by sex hormones in the breast epithelium (79). In relation to our present discussion on thyroid autoimmunity, a fascinating observation was made that the TSH-R and IGF-1R acted in concert regulating metabolism in thyroid [Reviewed in (80)]. The T and B lymphocytes have been found to overexpress IGF-1R in GD (81). The development of TAO has been speculated to be backed by the binding of IgG on IGF-1R (82). Excessive deposition of hyaluronan (hyaluronic acid, HA) in the extracellular matrix is one of the features of TAO. The fibroblasts in the orbit co-express TSH-R and IGF-1R that when activated by their agonists (TSH, IGF-1, respectively, and IgG), cause secretion of HA from these cells (83). Data on monoclonal antibody, M22 shows that it synergistically potentiates HA secretion after activating TSH-R and IGF-1R (84). The higher expression pattern of IGF-1R on T cells in GD exerts an antiapoptotic effect on the infiltrating lymphocytes causing their recruitment to the inflammation foci in the thyroid tissue thereby playing an important role in GD pathogenesis (85). The display of IGF-1R^+^ phenotype on B cells in patients with GD, dispenses high expansion of B cells and anti TSH-R antibody production (81). Pritchard et al. had enunciated the active role of IGF-1R as a self-antigen in the development of GD pathogenesis (86).

### Abrogation of Apoptosis in GD

The Fas-mediated apoptosis is considerably inhibited by TSH that results in hyperplasia of the thyrocytes in GD (87). The goiter phenotype in GD is strongly promoted by the inhibition of apoptosis mediated by Fas (88). Salmaso et al. showed that the thyroid infiltrating lymphocytes has higher expression levels of Fas/FasL that was synchronous with the significant apoptosis of the former in GD (89). The higher concentrations of IgG in GD have been known to mitigate the expression of Fas and at the same time potentiate the upregulation of Bcl-2 family of antiapoptotic molecules in the thyrocytes (90). However, the infiltrating lymphocytes are not spared by the upregulation of Fas expression and thereby undergo apoptosis augmented by the co-expression of proapoptotic molecules (91).

## The Case of HT

HT represents the most common chronic AITD with hypothyroidism responsible for significant morbidity among women (92). The subclinical presentation of HT is characterized by higher serum TSH levels. The disease manifests itself with overt loss of thyrocytes and circulating autoantibodies against Tg and TPO (93). HT has been studied to coexist with other autoimmune diseases in the same patient [Reviewed in (48)].

### Interplay of Immune Cells in HT

The thyroid gland cells show lymphocytic infiltration by T as well as B cells. Autoreactive T lymphocytes secrete various cytokines that exacerbate inflammation of intrathyroidal foci during autoimmune response (94). Stimulated by the inappropriate behavior of aberrant antigen presentation, uninterrupted invigoration of Th cells potentiates B cell activation that produces anti-TPO and anti-Tg autoantibodies. These autoantibodies prove the pathogenic role of B cells in thyrocyte damage. The B lymphocytes get activated upon recognizing a soluble autoantigen through their receptors on membrane, which is the membrane bound immunoglobulin (B cell receptor, BCR). This process is followed by two events: synthesis and secretion of specific autoantibodies by the activated B cells and B cells acting as APC to present autoantigens to CD4^+^ T cells that reciprocates this alliance through sustained B cell activation [Reviewed in (95)]. We have ample reports that advocate the presence of high titers of anti TPO autoantibodies in 90% of HT patients (96). Anti TPO autoantibodies assault thyroid cells via molecules of cytotoxic effectors (monocytes) and/or compliment activation (complement-mediated cytotoxicity, CDC) through the process of ADCC in HT (97). Additionally, the report of activated cytotoxic T cells resulting in thyrocyte destruction is quite evident in HT studies [Reviewed in (94)]. Apart from this, the ratio of Th1 to Th2 is elevated in cases of severe HT, where congruently high secretions of IFN-γ exacerbates the disease severity (98). The participation of Th17 in the pathogenesis of HT has been well-established. Li et al. showed significantly higher levels of intra-thyroid T17 cells and IL-17 serum in HT patients (99). Wang et al. have shown that increased concentration of Th17 cells in HT is associated with high CD4^+^ T cell-derived leptin levels (100). Leptin is an adipocyte hormone that shows high level structural similarities with IL-6, IL-11, IL-12 (101), and is a mediator of inflammation (102). High plasma leptin levels have been observed in women with postpartum thyroiditis and in postmenopausal women with HT (103).

Also, lower levels of Tregs have been documented in patients with HT (104). Its plausible to bring in the mention of one important protein, glucocorticoid-induced tumor necrosis receptor (GITR) in the case study of HT patients. GITR (a type 1 transmembrane protein) being expressed constitutively on Tregs, associates with its cognate ligand, GITR ligand (GITRL), present on APCs (105). The association of GITRL with GITR on Tregs causes cessation of immunosuppression by Treg thereby fuelling autoimmunity through the potentiation of Th17 cells (106). In a study on HT patient serum, augmented levels of GITRL correlated positively with higher concentrations of Th17 cells (104). CTLA-4 polymorphism has also been reported in HT (107). Thus, the infiltrating CD4^+^ T cells (self-reactive) engage the CD8^+^ cytotoxic T cells that kill thyrocytes through the secretions of perforin and granzyme (108). The activated B cells secret antibodies that lead to complement activation resulting in apoptosis of thyrocytes (11).

### Approval of Apoptosis in HT

The chronic state of ever deteriorating thyroid gland in HT majorly results from the Fas-mediated destruction of thyrocytes. Although, the Fas/Fas ligand (FasL) association initiates the destruction of thyrocytes, a remarkable study by Giorgio et al. showed the active role of infiltrating T lymphocytes in Fas-mediated cytotoxicity, where T cells actively expressed Fas and CD69. They showed that the T cells only expressed Fas but unlike thyrocytes, they did not show significant concentrations of FasL (109). The upregulation of Fas is strongly stimulated by the secretion of IL-1β by activated macrophages for proceeding toward thyrocyte destruction in HT (110). The destruction of thyrocytes in HT leads to the loss of thyrocytes and gradual accumulation of infiltrating monocytes causing diffused fibrosis (88). Bretz et al. advocates the expression of DR4, DR5 and TRAIL in thyrocytes that mediate apoptosis in HT (39).

The destruction of thyrocytes aided by all the above-mentioned plethora of molecular participants in the thyroid gland results in hypothyroidism.

## ER Stress in Thyroid Autoimmunity

Endoplasmic reticulum (ER), being the cellular repertoire of calcium, forms the pivot on which various cellular metabolic processes depend, aiming at the maintenance of homeostasis. The immune system as well as target tissue of AITDs, the thyrocytes, shares the characteristic of secretory cells with an extended ER.

### ER Stress in Autoimmunity

**Figure 3:**
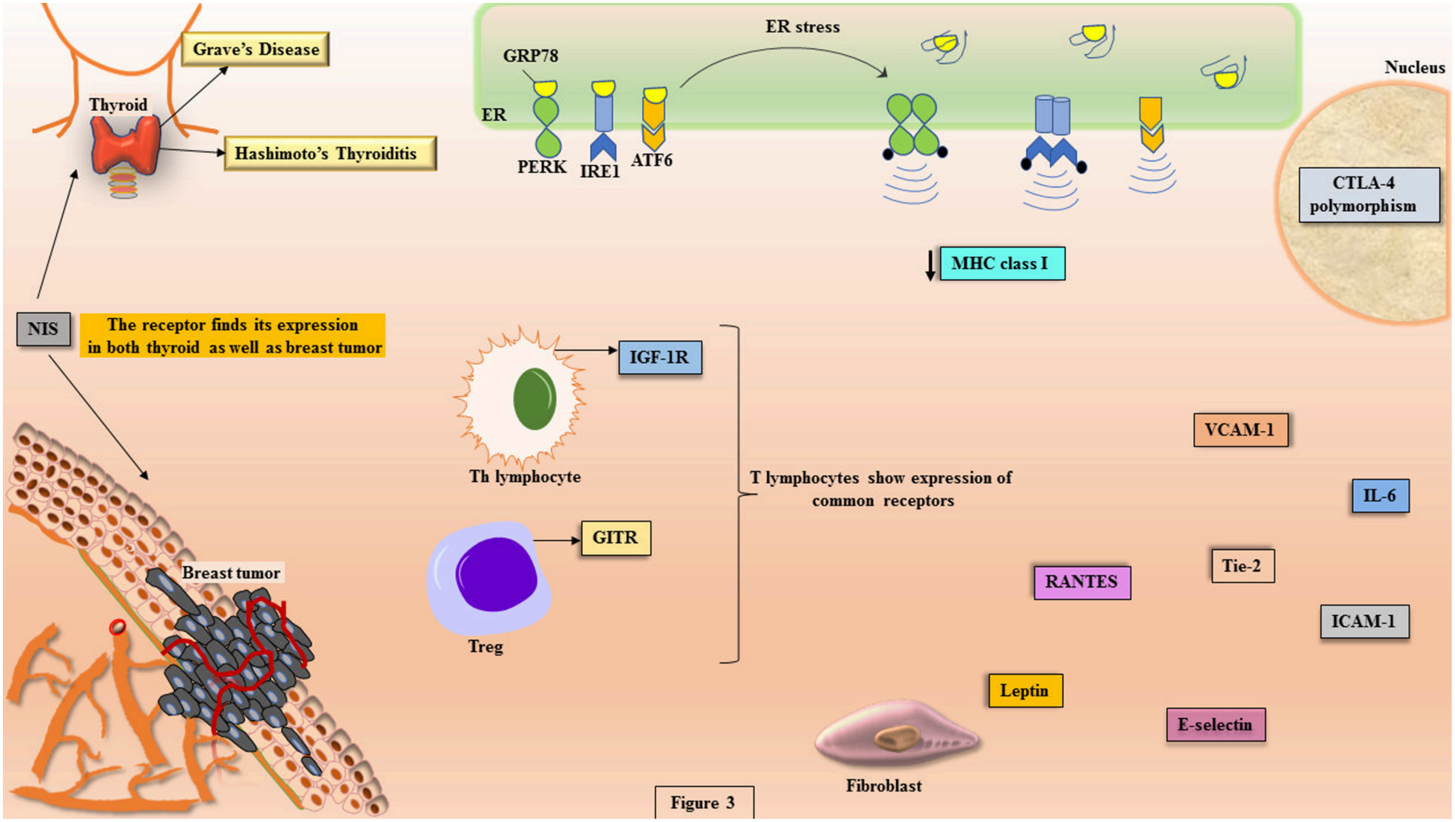
The potential molecules involved in the crosstalk between disease etiology of AITDs and breast cancer. The pathophysiology of AITDs and breast carcinogenesis display ER stress that shows the activation of UPR transducers (PERK, IRE1, and ATF6). In the manifest of AITDs, the MHC class I is downregulated that is the primary reason for the aberrant presentation of autoantigens while the same downregulation in breast cancer becomes reason for escape of tumor from the deleterious effects of cytotoxic T cells. Both the maladies display commonalities like: expression of GITR on Tregs, polymorphism in CTLA-4, illustration of IGF-1R on Th lymphocytes, higher concentrations of IL-6, RANTES, Tie-2, leptin, VCAM-1, ICAM-1, and E-selectin. In more than 80% of breast cancer tissues, the expression of NIS is highly significant, which is the primary iodide transporter in thyrocytes.

Under the conditions of imposed stress instigating AITDs, the function of ER in sustaining proteostasis gets disturbed that leads to ER stress. This initiates a cascade of signaling events called unfolded protein response (UPR) (Figure 3). The mammalian UPR showcases its signaling through three ER transmembrane resident proteins, PERK, IRE1, and ATF6, which get activated upon losing association of their ER luminal domain with the principal chaperone, BiP/GRP78 (111–115). PERK activation is demonstrated through its dimerization and trans-autophosphorylation of its cytoplasmic domain that further phosphorylates cytosolic translation initiation factor, eIF2α, triggering attenuation of translational machinery that is cap-dependent (116). This is followed by the translation of cap-independent, ATF4 mRNA that targets upregulation of UPR genes (117). The second sentinel of UPR, IRE1, upon activation shows its endoribonucleolytic activity by splicing XBP1 mRNA that gets translated into XBP1 transcription factor, which upregulates genes coordinating in degradation of misfolded protein and chaperone regulation (118). The translocation of XBP1 into the nucleus leads to the expression of UPR elements (UPREs). These are a group of genes that give assistance in protein folding and their further secretion, like proteins for ER-associated degradation (ERAD) (111, 112, 119). The activated ATF6 gets translocated to the Golgi apparatus, where it undergoes proteolytic cleavage giving another transcription factor, p50ATF6 that targets the genes for ER chaperones, XBP1 and CHOP (120).

Autoimmune inflammation has been studied to be induced by stress triggers and ER stress finds an important role in the pathogenesis of such autoimmune disorders (121, 122). The induction of ER stress paves the way to several pro-inflammatory immune arsenals like cytokines, IL-6 and IFNγ, which have been proven to be the key players in pathophysiology of autoimmunity (123, 124). A deranged ER homeostasis shows aberrant post-translational modifications (PTM) that have been studied to induce autoimmune response (125, 126). Autoimmune disorders register a conglomeration of ER stress-induced UPR activated molecules. The IRE1 mediates an immunogenic response to misfolded peptides, induces apoptosis resulting in the generation of auto-antigens that provoke autoantibodies and enhances the viability of cells showing autoreactivity (127). Autoreactivity to GRP78 has been observed in models of autoimmune disease (128). Approximately 80% of patients with autoimmune disease, rheumatoid arthritis, anti-GRP78 autoantibodies have been reported (129). It has been studied that BIP may function as an adjuvant that induces immune response under the conditions of ER stress (130). The role of PERK has also been studied in experimental autoimmune encephalomyelitis (EAE), where priming of UPR by IFN-γ leads to the activation of PERK that resists induction of disease (131).

### UPR in Pathogenesis of AITD

The break-down of self-tolerance in autoimmunity is majorly accounted for the accurate processing and presentation of peptides on MHC class I molecules. UPR plays an inevitable role in the processing of MHC class I peptide presentation during ER stress impinged by conditions of autoimmunity. It has been studied that induction of UPR impairs expression of MHC class I (132). Ulianich et al. have remarkably shown that the induction of ER stress in thyrocytes imparts diminishing effects on expression of MHC class I, and this accompanied the activation of NK cells with high levels of IFNγ (133). In the above discussion, we have seen that NK cells are actively involved in the pathogenesis of GD and HT (33). Ulianich et al. explained about the role of ER stress in the instantaneous downregulation of MHC class I molecules on thyrocytes and the relevance of IFN γ in the pathogenesis of AITD (133). Studies have reported about the aberrant expression of MHC class II by thyrocytes induced by IFN γ in the thyroid autoimmunity (57). Post-translational modification (PTM) of self-antigens in perpetuation of autoimmune diseases like rheumatoid arthritis (RA) and type 1 diabetes has been well-reported (134). The events of PTM on Tg has been suggested for the cryptic self in autoimmunity of thyroid (135). The innate immunity owes its proper functioning to UPR that supplies the demanding microenvironment with proinflammatory cytokines and chemokines. Among them, a remark on IL-6 is appealing here because the signal transducers of UPR have been observed to elicit the expression of IL-6 (136). On the same note, IL-6 has been studied to show effect in the development of TAO (137).

The very specialized function of thyrocytes is essential in the secretion of thyroid hormones Tg, T4, and T4, which relies substantially on the proper functioning of the ER. Hence, stimuli causing ER stress paves the way to UPR in thyrocytes (138). Administration of iodine in GD patients has been shown to impose stress thereby diminishing the expression of MHC class I as well as class II molecules in thyrocytes, which ultimately facilitate the presentation of faulty autoantigens (139). In most of the AITDs, Tg is the major target of autoantibodies because of its unusual processing in the ER (8). The influence of ER stress in autoimmunity is well-described in various reviews (126). However, the participation of UPR sentinels, PERK, IRE1 and ATF6 have not been extensively studied in the cases of AITDs.

## Why is the Female Sex More Prone to Autoimmunity: the Gene Connection?

We have accumulating reports that attempt to explain the disparity in the preponderance of AITD in females (140). The **a**uto**i**mmune **re**gulator (AIRE) gene finds its expression in thymus, lymph nodes and fetal liver, which are the workshops for T cell maturation [Reviewed in (141)]. AIRE codes for a transcriptional regulator that is a significant mediator of the central tolerance working in sync with Tregs for preventing autoimmunity [Reviewed in (141)]. The etiology of autoimmune polyglandular syndrome (APS) type 1 uncovers its manifestation in an autosomal recessive mutation in the AIRE gene due to which the patient suffers from Addison's disease and thyroid autoimmunity [Reviewed in (142)]. Under the effect of AIRE mutation, the self-antigens do not undergo negative selection/clonal deletion that fallouts as autoimmunity. Latest studies have shown that sex hormones are involved in the thymic AIRE regulation, for example, androgens heighten the negative selection of thymocytes through AIRE upregulation thereby conferring protection against autoimmunity, whereas estrogen downregulates expression of AIRE leading to the flaring of autoimmunity (143). Also, an increase in the testosterone levels in females suffering from polycystic ovary syndrome heightens their chance of getting AITDs (144). A multitude of female patients with AIDT show higher B cell antibody production (145). Moreover, there are reports of skewed X- chromosome inactivation (XCI) (same X-chromosome inactivation in ≥ 80% of cells) in women manifesting the AIDTs: skewing odd ratio of XCI with GD was 2.54 and with HT it was 2.40 (11).

## The Alliance Between AITD and Breast Cancer

Accumulating reports unequivocally corroborate the association between AITD and breast cancer (146). Nearly every type of thyroid malaise has been witnessed to be cohesive with the breast malignancy like, nodular hyperplasia and hyperthyroidism (147). The elaboration of breast cancer in patients with GD as well HT has been well-documented (148). Smyth et al. advocated the presence of considerable concentrations of anti-TPO autoantibodies among patients with breast cancer (149). Not only this, the presence of anti-TPO autoantibodies give a significant prognostic advantage for breast cancer (150). The anti-TPO and anti-Tg autoantibodies have been given their roles as potential protective agents against breast carcinoma in hypothyroidism (11). On the other hand anti-TSH-R autoantibodies have been reported to have associative relationship with the risk of breast cancer (151). The presence of TSH-R in mammary epithelial cells is well-acknowledged (152).

### Breast Carcinoma and Its Prevalence in Patients With AITDs

Breast cancer represents heterogeneity in its molecular as well as histological manifest. Based upon the expression of hormone receptors, the subtypes of breast carcinoma are as follows: either estrogen (ER) or progesterone (PR) receptor positive (ER/PR^+/−^), neither ER nor PR positive (ER/PR^−/−^), HER2 positive (HER2^+^) (they show overexpression of oncogene ERBB2) and devoid of any receptors called triple negative (TNBC). Hormonal therapy works for ER^+/−^//PR^+/−^ breast cancers whereas anti-HER2 therapy is used effectively for HER2^+^ subtypes. TNBC being most aggressive of all is linked with higher relapse rate (153).

We have accumulating reports on the association amid autoimmune thyroid diseases and breast cancer (5). Various research findings have augmented the data on the prevalence of thyroid autoantibodies in breast cancer patients (154). We have studies that show that HT patients are at higher risk for breast cancer (155), however discrepant data also shows non-approval of relationship between HT and breast cancer (156, 157) In one study group of breast cancer patients, thyroid antibodies were frequently reported with the manifest of HT. This study advocates the prevalence of cytotoxic antibodies in breast cancer patients (148).

### The Enigmatic Role of NIS

The expression of NIS in the mammary gland epithelium is prominently evidenced during lactation as well as breast malignancy (158). Reports demonstrate that in more than 80% of breast cancer tissues, the expression of NIS is highly significant (159), nonetheless, this expression did not show any meaningful iodide uptake (160). Experiments have proven that *in vitro* stimulation of NIS expression in breast tissue can be attained by lactogenic hormones and insulin (161). The expression of NIS in the thyroid gland is stimulated by TSH whereas its expression is only transiently dependent on the TSH activity during the period of lactation in the mammary gland (162). Albeit, the expression of NIS in some breast cancer cells have been significantly reported to be induced by retinoic acid (163). The molecular journey of the regulatory cascade culminating in the NIS expression is quite appealing. The most effective TSH-responsive enhancer element is present in the NIS promoter (chromosome 19), which is known as the NIS upstream enhancer (NUE). The cofactor responsible for suppressing NUE is PBF (a proto-oncogene), which is pituitary tumor-transforming gene-1 (PTTG1)- binding factor, has been documented to co-localize with cytoplasmic NIS [Reviewed in (164)]. In some cases of differentiated thyroid cancer, the transcriptional regulatory network for NIS expression fails, due to which thyroid cancer tissues show mitigated levels of NIS mRNA expression [Reviewed in (164)]. Contrary to this, studies also report that these differentiated thyroid cancer tissues showed copious cytoplasmic NIS expression rather than cell surface expression. This observation is in coherence with the retention of NIS in the cytoplasm rather than their surface expression on the breast tumor. This has been a proposed mechanism for the diminished radioiodide uptake by both thyroid as well as breast cancers [Reviewed in (164)]. Most thyroid and breast cancers have been shown to abundantly express PBF, which is possibly responsible to retain NIS in the cytoplasm [Reviewed in (164)]. This PBF mediated mitigation in the functional expression of NIS in the thyrocytes, has been linked to the enlargement of thyroid in transgenic mouse model (165).

The upregulation of NIS expression and at the same time, their intracellular retention in various other cancers like stomach and liver in addition to thyroid and breast carcinoma, suggests about a common molecular link. In a remarkable study, the non-solute transport role of NIS was delineated, where it was proved that there is an interaction of NIS with the leukemia-associated RhoA guanine exchange factor (LARG) leading to the activation of RhoA [Involvement of RhoA in breast cancer is discussed elsewhere (166)], which supports cell migration (167).

The discussion on NIS would be incomplete here without mentioning about the anti-NIS autoantibodies. As reviewed, reports have endorsed anti-NIS autoantibodies in AITDs but we are in dearth of successful data that can prove the role of these autoantibodies in the autoimmune thyroid pathogenesis (168).

### The Molecules Upregulated/Downregulated in AITD May Foster Breast Cancer

The malignancy in breast is dependent on the hormonal activity (169). Being women is one of the greatest risk factors for the development of breast cancer (170). The various epidemiological and molecular studies discussed herein do correlate the existing alliance between high prevalence of AITD in women and breast cancer. The molecules participating in the sustenance of autoimmunity in AITD share functional relevance in the development and progression of breast cancer. We present here an analysis on the gamut of molecules that arise during the pathophysiology of AITDs, which can be a potent prop for breast neoplasia.

### The Immunological Panorama

The breast carcinoma shows unusual quantities of stroma with fibroblasts, vascular tissues and lymphocytic infiltration that are implicated in the development of cancer (171). The tumor microenvironment harbors several immune cells that turn foe to the host whereby they provide a helping hand for the establishment of tumor. Numerous tumor-infiltrating lymphocytes sustain the growth of tumor by the suppression of host immunity by using their arsenals like Tregs, myeloid-derived suppressor cells (MDSCs) and macrophages (172). Under the conditions of perturbed homeostasis due to the stress imposed by coexisting diseases like AITDs, the various cytokines, chemokines, and extracellular matrix molecules secreted during the pathogenesis of AITD might aid in the recruitment of molecules that potentiate cancer. The statistical analysis shows that the exhibition frequency of cancer is higher in tissues under the assault of chronic inflammation (173).

The cellular immune responses triggered by MHC class I molecule are very important for intracellularly processed peptides and this owes its regulation to ER proteostasis. We have ample studies that support the idea of dependence of MHC class I molecule expression on the hormonal modulation in a specific tissue (174). The characteristic feature of declined expression of MHC class I molecules on CD4^+^ T cells in patients with autoimmune diseases is in congruence with the similar pattern observed in malignancy (175). A failure in proper regulation of self-antigen presentation on MHC class I molecule in thyroid paves the way to AITD (174). Analogously, it has been shown in HER2-overexpressing breast cancer patients that the cancer escapes from CTL through MHC class I downregulation (176).

Metastasis showcases its protection against NK cells and CTL through the alteration of FoxP3^+^ Tregs under the influence of Bregs, in breast cancer (177). In mice model of breast cancer, the MDSC are known to be educated by Bregs that render the former pro-metastatic. MDSCs represent a heterogenous group of cells that have the power to restrain function of T cells thereby disseminating pathogenesis of cancer (178). The activity of MDSCs has been known to be activated by both IFNγ as well as TGFβ, and in cancer models their build-up is potentiated by IL-6 and IL-1β (179). Chen et al. showed that CTLA-4 not only negotiated the downregulation of MHC class II expression but also the activation of T cells, resulting in the perpetuation of tumor proliferation (180). In a meta-analysis study, it has been suggestively disclosed that polymorphism in CTLA-4 is related with the susceptibility of breast cancer (181). A further addition in the similarity event amid AITDs and breast cancer is the presence of Tregs expressing GITR. Krausz et al. have proven that the expression of GITR on Tregs is associated with rise in metastatic potential of breast carcinoma (182).

The performance of IGF-1R is critical in the growth and migration of cells, therefore it is not startling that the signaling of IGF-1 strongly props cancer establishment. Reports show that ~50% of breast carcinoma manifest IGF-1R expression (183). Astonishingly, the abundance in the availability of IGF-1 and IGF-2 accentuate the tumor microenvironment, which favors the unimpeded migration of breast cancer (184). Hence reports of IGF-1R activity in GD strongly correlates with the addition of these cancer potentiating molecules in the cellular microenvironment.

Aforementioned role of IL-6 in the inflammatory events of TAO finds its similar function in the pathophysiology of breast cancer where IRE1 arm of UPR elicits inflammatory molecules (NF-κB) through IL-6 (112, 185). The boosting concentrations of IL-6 during the establishment of GD pathogenesis, might act as host factors that can support breast carcinoma. GD registers a comparative resistance to apoptosis owing to its distinct cytokine expression profile, where RANTES has been witnessed to support lymphocytic infiltration in the thyroid. Analogously, breast cancer progression has been strongly propped by the elevated expression of RANTES (186). Another important pro-inflammatory molecule associated with autoimmunity is leptin (187). Niu et al. showed through meta-analysis that leptin influences the development and progression of breast cancer (188). We have accumulating reports from studies in breast cancer cell lines that show the active participation of leptin in proliferation and anchorage-independent propagation of breast tumor (170).

### Adhesion Molecules Are the Surrogate Players

The adhesion molecules have been studied to act as the potential markers of the autoimmune manifest, which play crucial role in the immunomodulation of the patient (189). These molecules are vital in the interaction of cell to cell as well as cell to its basement that has been postulated to be pivotal in inflammation and metastasis (190).

The expression pattern of adhesion molecules in the vasculature depends on the cytokines of pro-inflammatory and pro-angiogenic stimulus that we have seen from the above discussed data on the thyroid autoimmunity. The activated endothelial cells secrete adhesion molecules like VCAM-1 and E-selectins (191). These adhesion molecules have been verified to promote inflammation in the endothelium that facilitates metastatic seeding in distant organs (192). Higher serum concentrations of soluble VCAM-1 (sVCAM-1) and ICAM-1 have been reported in breast cancer (193). The higher expression levels of E-selectins were also observed in the endothelium of breast cancer (194). Byrne et al. showed that significant levels of serum VCAM-1 closely associate with tumor angiogenesis (191). Apart from the tumor cells, the host cells also recruit factors that support angiogenesis (neovascularization), which include molecules sourced from endothelial cells, extracellular matrices, fibroblasts and platelets (195). The higher levels of E-selectin in patients with GD, helps in the invasion of cells affecting the dissemination of autoimmunity (196). The expression of Tie-2 has also been well-reported in breast cancer that supports angiogenesis (197). Thus, the microenvironment created by a surplus of adhesion molecules due to the establishment of thyroid autoimmunity might play a role in augmenting breast cancer progression.

The pictorial representation of this molecular crosstalk has been illustrated in Figure 3.

### The Key Players of ER stress

Under the conditions of imposed ER stress, the cells and their environing extracellular surroundings are under constant pressure to discern life or death decision. Breast cancer is one good example of this situation. We have learnt from various studies that ER stress and hence the ensuing UPR plays major role in the pathophysiology of breast carcinogenesis (112). Young et al. have shown increasing levels of ER stress in thyroid tissue causing autoimmune diseases (198). In congruence with the aforesaid impairment of MHC class I expression due to UPR induction in AITDs, it is well-established that diminishing levels of MHC class I in breast tumor is an important parameter for escape from the effects of CTL (199). In a study by Inoue et al. it has been well-documented that HER2 signaling mitigates the expression of MHC class I on breast cancer cells (176). Apart from acting pro-survival to tumors, UPR shoots up the concentration of inflammatory molecules that further bolster the progression of tumor. The UPR sculpts cancer proliferation through the cytokines like IL-6, released by the tumor infiltrating lymphocytes (200, 201). A study on TNBC cells showed that the IRE1 axis of UPR activated the production of IL-6, which was pro-tumorigenic (201). Additionally the IRE-1/XBP1-mediated upregulation of inflammatory molecule, NF-κB has been documented in studies on breast cancer (112). Another UPR marker, PERK, show expression in activity hinting about the crosstalk between AITDs and breast cancer. Human breast ductal carcinoma shows phosphorylation of PERK thereby marking the active involvement of UPR signal transducers (202). Chen et al. have shown that XBP1 silencing effectively diminished growth of mammosphere in TNBC cell lines (203, 204). Interestingly, Bartkowiak et al. have reported the activation of UPR in the disseminated tumor cells in the bone marrow of breast cancer patients (205).

These are the key signatures which are common to the pathophysiology of both AITDs and breast cancer. Therefore, a radical approach to develop molecules against the aforementioned UPR signatures can be a potential therapeutic intervention to tackle both the diseases.

## Future Perspective

The development of cancer is not only pronounced by the intrinsic autonomous biochemistry operating in the tumor but also the changes in the neighborhood of the primary tumor niche, which involves alterations in the concentrations of various molecules beyond their homeostatic levels. The pathophysiology of AITDs strongly hints that there is a significant upsurge in the concentration of various molecules that cross the boundary of one endocrine organ (thyroid) to another (breast). We have witnessed a distinctive collaboration among molecules showing heterotypic association amid the AITD manifest and the incipient breast tumor, which is remarkable. Despite the developments in the treatment of autoimmune disorders using immunomodulatory molecules, the results are not always satisfactory. Hence the knowledge about key molecules involved in the development of autoimmunity with special emphasis on stress-induced signaling molecules like the UPR markers is a mandate. Targeting the molecules involved in ER stress can help alleviate the destruction caused by the possible crosstalks between AITDs and breast cancer. For instance, in some models of autoimmune disorders, the IRE1 inhibitor has come out as a potential therapeutic candidate (206, 207), therefore this gives a good idea to extrapolate the study in AITDs. In the same note of finding clue to the crosstalk, some of the unventured areas worth raising at this point are discussed herein.

The glycosylation status of autoantibodies, especially IgG is important in the pathophysiology of autoimmune diseases (208). ER being the primary cellular compartment orchestrating the PTM of nascent peptides, becomes an important site for glycosylation of autoantibodies. The cellular receptors for the constant domain of IgG (Fcγ receptors, FcγR) act centrally in the mediation of inflammation caused by autoantibodies (209). Interference in the glycosylation pattern on Fc portion can malign the situation resulting into autoimmunity response by autoantibodies (208). Aberration in the functioning of glycosyltransferases leading to remodeling of glycosylation status on IgG, has been uncovered in patients with rheumatoid arthritis (an autoimmune disease) (210). Analogous to this, the cancer metastasis has been extensively studied with respect to aberration in the glycosylation pattern of various immune as well as adhesion molecules (211). The genetic dysregulation pertaining to crucial enzymes of glycosylation like Mgat3 or Mgat5, play significant role in cancer progression. In one remarkable study, the ER stress inducing agent, Tunicamycin, has been shown to interfere protein glycosylation whereby it reduced angiogenesis in breast tumor models (212). But we are in dearth of such experiments pertaining to AITDs. Therefore, studies unraveling any potential aberration in glycosylation in the cases of AITDs is strongly demanded, which can help us join some of the missing links in the crosstalks amid thyroid autoimmunity and breast cancer.

The role of Galectin-9 (Gal-9), which is a glycan-binding protein, in the innate as well as adaptive immune response is very pertinent. Gal-9, being one of the prime regulatory molecules acting against inflammation, works by downregulating the Th1 and Th17 response (213). Leskela et al. have shown that the expression of Gal-9 was diminished in peripheral blood samples of GD but not HT patients and through functional assay they showed that exogenous Gal-9 imposed the downregulation of Th1/Th17 response (214). In the same note, Irie et al. have shown that the expression level of Gal-9 is low in breast cancer. They further revealed through ectopic expression of Gal-9 in MCF-7 cell lines that Gal-9 displayed anti-metastatic potential (215). Hence, the role of Gal-9 in GD needs further exploration so that the link to breast cancer can be deciphered.

The significance of concomitant occurrence of polymorphism in the CTLA-4 gene, reported in both AITDs and breast cancer, need further explorations on its functional front. Studies have proven that the blockage of CTLA-4 not only recovers the T cell activity but also mitigates breast cancer pursuit, whereby therapeutic targeting of CTLA-4 would add up a step in the therapy of breast cancer (180). Accordingly, venturing more into the activity of CTLA-4 during the pathogenesis of AITDs can help in further understanding of the potential molecular targets for therapy. Furthermore, the available agonists of GITR focusing on its translational modulation in cancer have been reviewed elsewhere; hence a trial of the same in AITDs would help in accentuating our attempts of catching up with the knowledge lag in AITD pathophysiology (216). Another striking molecule under consideration for therapeutic arbitration is IGF-1R, which has been subjected to blockage by several agents as discussed elsewhere (184). The abrogation of Fas-mediated apoptosis is well-cited in the thyrocytes of GD patients. Parallelly, breast tumor exhibits abolition of Fas-mediated apoptosis, as shown by the study of Radin et al. (217). The authors have extended their study to show that the inhibition of antiapoptotic Fas antagonizing protein, Lifeguard (LFG), whose expression level shoots up in TNBC cell line can increase the chemotherapy efficacy (217). Therefore, studies on LFG activity in AITDs might unearth any potential role of this molecule in the pathophysiology of the disease.

The past two decades has witnessed tremendous research on the underlying mechanism of AITDs. Accumulating data have exhibited insights into the dynamism of immune cells in development and progression of cancer. Experiments involving the pharmacological activation of ER stress in cancer cells reported about the secretion of some soluble factors that led to the induction of not only UPR markers but also pro-inflammatory cytokines in the macrophages (218). This phenomenon of “transmissible ER stress” affected APCs and induced expression of immunosuppressant (219), thereby attesting one major observation that cancer cells under ER stress cleverly modulate immune cells through the secreted factors. Therefore, looking into the intricacies of these soluble factors can help in understanding if the same immunomodulation is happening in the pathophysiological relationship of AITDs and breast cancer. Both, AITDs as well as tumorigenesis largely depend upon an orchestrated interplay displayed by the various molecules that are categorically defined as being either cytokines or receptors or adhesion molecules or autoantibodies. The three sensors of UPR, PERK, IRE1 and ATF6, show a complex entwinement of highly coordinated signaling pathway that regulates the proper functioning of secretory cells (thyroid and breast). An interference at the working of these molecules can be a potential strategy that can aim at impeding the progression of maladies associated with AITDs and breast cancer. At this point of time, our science demands more reductionist approach to the reconceptualization of molecular crosstalks that ease the development and establishment of one disease by another. Lots of epidemiological data exists that prove the association of AITDs and breast cancer but at the same time we lack the compendium that can elucidate the reason behind such alliance. We need more AITD culture studies focusing on target molecules of UPR that can be replicated in animal models to give a comprehensive proof of the molecular crosstalks that seem to build a network connecting two organ pathologies. This will not only present a holistic lucidity of the underlying mechanisms but also help in rationalizing unrecognized prognostic markers, which might be common to both AITDs and breast cancer.

## Author Contributions

SR and RM conceived the idea. SR, ATJ, AJ, DD, AS, JK, and RM contributed to the writing of manuscript. SR, ATJ, AS, JK, and RM contribute to the proof reading, editing and figure preparation for the manuscript.

### Conflict of Interest Statement

The authors declare that the research was conducted in the absence of any commercial or financial relationships that could be construed as a potential conflict of interest.
